# Toward a quantitative description of solvation structure: a framework for differential solution scattering measurements

**DOI:** 10.1107/S2052252524003282

**Published:** 2024-05-01

**Authors:** Niklas B. Thompson, Karen L. Mulfort, David M. Tiede

**Affiliations:** a Division of Chemical Sciences and Engineering, Argonne National Laboratory, 9700 South Cass Avenue, Lemont, Illinois 60439 USA; UCL, United Kingdom

**Keywords:** aperiodic structures, high-energy X-ray scattering, inorganic chemistry, intermolecular interactions, pair distribution function analysis, SAXS, WAXS, HEXS

## Abstract

Appreciating that the role of the solute–solvent and other outer-sphere interactions is essential for understanding chemistry and chemical dynamics in solution, experimental approaches are needed to address the structural consequences of these interactions, complementing condensed-matter simulations and coarse-grained theories. High-energy X-ray scattering (HEXS) combined with pair distribution function analysis presents the opportunity to probe these structures directly and to develop quantitative, atomistic models of molecular systems *in situ* in the solution phase. However, at concentrations relevant to solution-phase chemistry, the total scattering signal is dominated by the bulk solvent, prompting researchers to adopt a differential approach to eliminate this unwanted background. Though similar approaches are well established in quantitative structural studies of macromolecules in solution by small- and wide-angle X-ray scattering (SAXS/WAXS), analogous studies in the HEXS regime—where sub-ångström spatial resolution is achieved—remain underdeveloped, in part due to the lack of a rigorous theoretical description of the experiment. To address this, herein we develop a framework for differential solution scattering experiments conducted at high energies, which includes concepts of the solvent-excluded volume introduced to describe SAXS/WAXS data, as well as concepts from the time-resolved X-ray scattering community. Our theory is supported by numerical simulations and experiment and paves the way for establishing quantitative methods to determine the atomic structures of small molecules in solution with resolution approaching that of crystallography.

## Abstract 2

Total X-ray scattering measurements of dilute solutions are uniquely well suited to address the structural basis for the effects of solvation, exemplified by the success of small-/wide-angle X-ray scattering studies of macromolecules in solution. Herein, we extend this methodology to the high-energy X-ray regime by developing a theoretical framework to describe the differential solution scattering experiment, supported by numerical simulation and experiment.

## 1. Introduction

Solvation is a ubiquitous phenomenon that profoundly affects the physiochemical properties of molecular systems. Underlying these effects is the ‘solvation structure’, including how dissolution affects the atomic structure of the solute as well as the way in which the solvent is ordered locally about the solute. Even in the simple case of outer-sphere self-exchange, the organization of solvent about the exchanging ions makes a decisive contribution to the rate of electron transfer, as eloquently expounded by the work of Marcus (1993 ▸). Despite the long history of Marcus theory, it is only with the recent advent of the X-ray free electron laser (XFEL) that experimentalists have succeeded in detecting the structural rearrangement of the solute–solvent interface at the ultrafast timescale of photoinduced electron dynamics (van Driel *et al.*, 2016 ▸; Khakhulin *et al.*, 2019 ▸; Biasin *et al.*, 2021 ▸; Katayama *et al.*, 2023 ▸). More broadly, synthetic chemists have long sought to engineer the outer coordination spheres of transition metal complexes to engender favorable solvation interactions in catalyst and device design, often taking inspiration from natural metalloenzymes (Drover, 2022 ▸; Ghosh *et al.*, 2022 ▸). Likewise, manipulating the solvation structure of cations in electrolyte is an area of emerging importance in battery research (Rajput *et al.*, 2018 ▸; Cheng *et al.*, 2022 ▸).

Although controlling solvation structure and dynamics remains a key objective in chemical research, experimental approaches to resolve such structures at the atomic scale are underdeveloped. Spectroscopic techniques such as nuclear magnetic resonance (Clore & Gronenborn, 1991 ▸; Bifulco *et al.*, 2007 ▸), electron paramagnetic resonance (Attanasio, 1986 ▸; Mustafi & Makinen, 1988 ▸), vibrational (Perera *et al.*, 2008 ▸; Sun & Petersen, 2017 ▸) or X-ray absorption spectroscopy (Chen, 2004 ▸; Penfold *et al.*, 2014 ▸; Jay *et al.*, 2020 ▸) can provide valuable insights into the effects of solvation, but remain indirect measures of structure. The extended X-ray absorption fine structure region of *K*-edge X-ray absorption spectroscopy data—a type of scattering phenomenon—can provide direct structural information regarding molecules in liquid or frozen solution; however, typically only the first coordination shell of the absorbing atom can be resolved (Filipponi, 1994 ▸; Garino *et al.*, 2014 ▸).

In contrast, total scattering measurements provide two-body information out to arbitrary distances with a signal that, in principle, decays only as a result of the actual disorder in the system (Egami & Billinge, 2012 ▸; Garino *et al.*, 2014 ▸), and are thus uniquely suited to address solvation structure (Hertz, 1970 ▸; Soderholm *et al.*, 2005 ▸; Megyes *et al.*, 2008 ▸; Mulfort *et al.*, 2013 ▸; Scalambra *et al.*, 2021 ▸). Combining large two-dimensional area detectors with the high-energy incident radiation available at third-generation synchrotron light sources (*h*ν > 50 keV or λ < 0.25 Å), it is now possible to routinely collect total X-ray scattering measurements out to large momentum transfers, *Q*_max_ ≥ 20  Å^−1^, where *Q* = 4πsin(θ/λ) for photons scattered elastically at an angle of 2θ (Billinge, 2019 ▸). Given the duality between reciprocal space (*Q*) and real space, such measurements yield structural information with sub-Ångström spatial resolution, as is typical in crystallographic analysis. However, while crystallography relies on the presence of Bragg diffraction from a crystalline sample, it is always possible to analyze the total scattered intensity from samples in arbitrary phases of matter.

In the context of total scattering experiments on solutions, where the objective is to determine the solvation structure of, for example, a small molecule, a practical limitation arises from the fact that the scattering signal from the bulk solution dominates the signal from the solute, the latter being proportional to its volume fraction in solution. To address this, a differential approach is typically adopted. That is, two measurements are made, the first of the solution itself, and the second of an otherwise identical sample lacking the solute of interest. Then, adopting the practice employed in small-angle/wide-angle X-ray scattering (SAXS/WAXS) studies of macromolecules (Jeffries *et al.*, 2021 ▸), the second measurement is treated as an experimental background and subtracted from the first, with the aim of isolating the solute signal from the bulk solvent signal. Although a theory for subsequent quantitative, atomistic structural modeling has been investigated in the case of SAXS/WAXS data (Park *et al.*, 2009 ▸; Köfinger & Hummer, 2013 ▸; Chen & Hub, 2014 ▸), where *Q*_max_ ≲ 1 Å^−1^, such treatment is lacking for the case of total scattering data collected in the high-energy regime.

Indeed, unlike in the SAXS/WAXS setting, where spatial resolution is limited, high-energy X-ray scattering (HEXS) measurements are typically transformed to a pair distribution function (PDF) to extract quantitative structural information in real space. Quantitative information (*e.g.* electron densities, bond length distributions *etc.*) can be measured directly from the transformed data when a single peak or a multiplet can be reliably assigned to a specific correlation (Neuefeind *et al.*, 2004 ▸; Soderholm *et al.*, 2005 ▸; Skanthakumar *et al.*, 2017 ▸). However, experimental PDFs often present a complex convolution of many signals, which can be difficult to assign *a priori* without a detailed and realistic structural model for comparison. Nevertheless, we have employed differential HEXS measurements to assign the solution structures of molecular electrocatalysts, generated *in situ* (Yang *et al.*, 2016 ▸), and to characterize the atomic scale differences of the intramolecular structures of transition metal complexes between the solid and solution phase (Xie *et al.*, 2022 ▸). These studies relied on quantum-mechanical models [*i.e.* density functional theory (DFT) calculations] to predict and assign structural features in the experimental PDFs, with the limitation that any intermolecular (*e.g.* solute–solvent) correlations are neglected.

As we will show, the development of quantitative models of such data requires added complexity, including important contributions to the differential scattering arising from solute–solvent and solvent–solvent pair correlations. The former have been investigated in detail in the context of time-resolved X-ray solution scattering (Kim *et al.*, 2009 ▸; Ihee, 2009 ▸; Haldrup *et al.*, 2010 ▸); however, the principal solvent–solvent correlations differ in origin between the static and time-resolved settings (see below). In this contribution, we present a theoretical framework for the static differential solution scattering experiment from first principles within kinematic scattering theory. Our aim is twofold: first, to clearly delineate the information content contained in the differential scattering intensity and second, to develop a formalism analogous to standard PDF analysis that is applicable to quantitative structural studies of small molecules in solution.

## 2. Theory

### 2.1. Standard total scattering formalism

From a structural perspective, the information content contained in the total scattering measurement is encoded by the coherent interference of photons scattered elastically from distinct pairs of atoms within the sample, the coherence volume of the X-rays being typically microscopic (Grübel & Zontone, 2004 ▸). The standard formalism for total scattering structural studies involves the Fourier sine transform pair 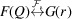 , referred to as the ‘reduced total scattering structure function’ and the ‘reduced PDF’, respectively (Egami & Billinge, 2012 ▸).

Consider the scattered intensity from a collection of 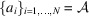 atoms within the coherent scattering volume *V* of the incident X-rays. Within kinematic scattering theory under the independent atom approximation, it is usual to define 

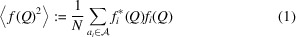

and 

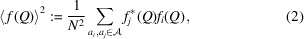

where *f_i_* (*Q*) is the (in principle, complex-valued) atomic form factor of atom *a_i_* (*i.e.* the Fourier transform of its associated electron density). Equation (2) defines the average scattering power ‘per atom’ (Guinier, 1994 ▸). If the total illuminated volume is larger than the coherence volume of the incident X-rays, and the coherence length of the scattering object is smaller than this coherence volume, then the observed scattering will be azimuthally isotropic, and the total coherent scattering intensity is given by the Debye scattering equation

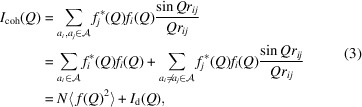

where *r_ij_* is the distance between atoms *a_i_* and *a_j_*. In the second equality, we have separated out the terms in the double summation for which *i* = *j*—giving the self-scattering intensity, *N*〈*f* ^2^〉—and for which *i* ≠ *j*—giving the so-called distinct scattering intensity *I*_d_.

Only the latter term encodes the two-body information, so the reduced total scattering structure function, *F*(*Q*), is simply the *Q*-weighted distinct scattering, normalized to the average scattering power of the sample, which is dual to the reduced PDF, *G*(*r*), under the Fourier sine transform (Farrow & Billinge, 2009 ▸) 

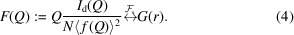

Although *F*(*Q*) and *G*(*r*) encode the same structural information, the PDF admits an intuitive interpretation, as it is essentially a weighted histogram of all the atomic pair distances in the sample.

### 2.2. Total scattering formalism for dilute solutions

**Figure 1:**
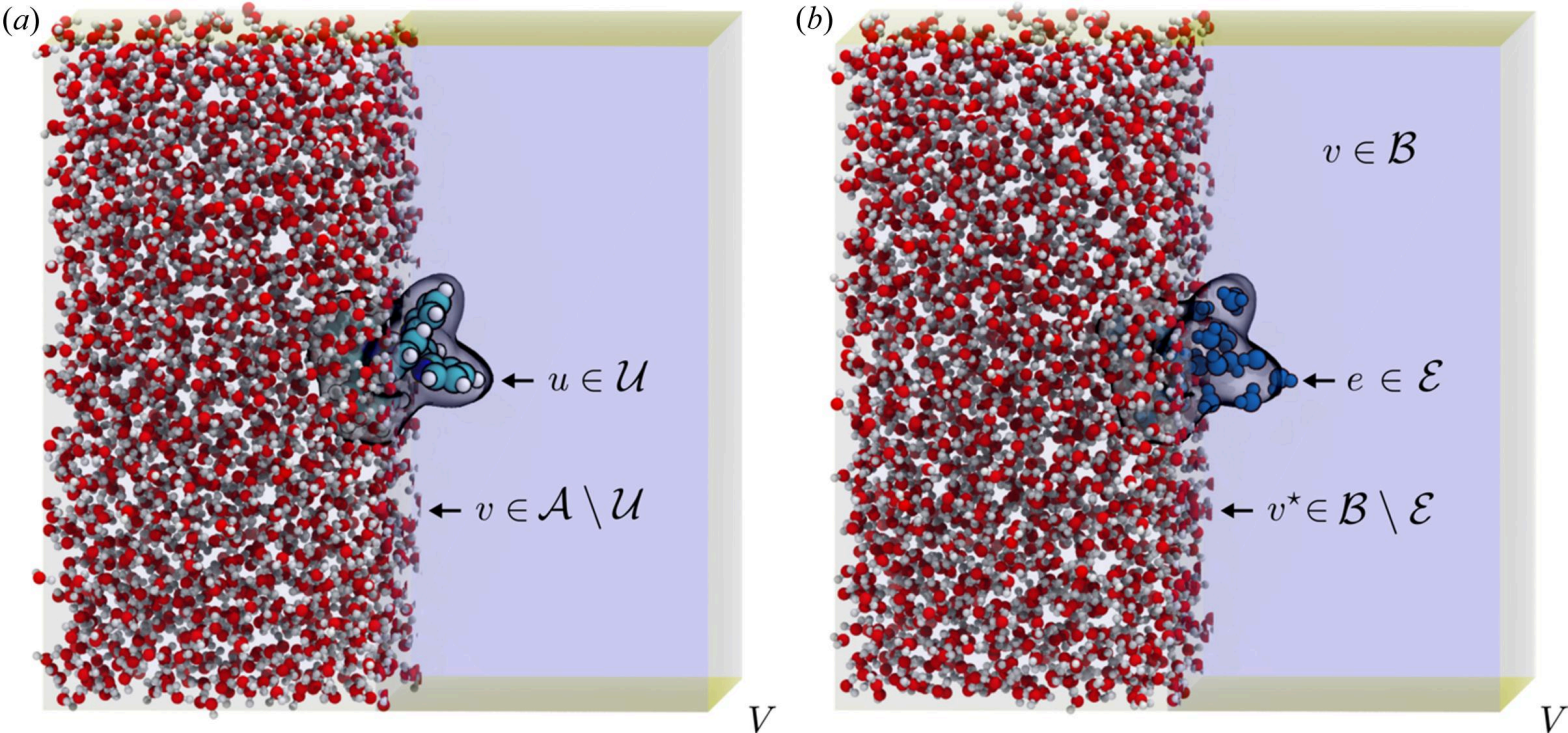
Description of the solution scattering experiment. (*a*) Schematic of the liquid unit cell *V* of sample A. The gray surface shows the van der Waals volume of the solute, defining the excluded volume. (*b*) Schematic of an equivalent volume *V* of sample B, showing how the excluded volume defines a droplet of solvent atoms (shown in blue). For clarity, only a half of the solvent atoms outside the excluded volumes are rendered.

In the differential solution scattering experiment, we perform two measurements. In measurement A [Fig. 1 ▸(*a*)], we collect the total scattering from a dilute solution (*e.g.* where the solute is a molecule); the second measurement B [Fig. 1 ▸(*b*)] is performed on an otherwise identical sample, lacking the solute (*i.e.* pure solvent plus any other constituents from A). Let *V* now denote the liquid unit cell of the solution, that is, the minimal volumetric unit of composition of the homogeneous solution (Haldrup *et al.*, 2010 ▸), and 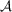 denote the collection of atoms contained within this volume. We assume that the coherence volume, integrating over many liquid unit cells *V*, is identical in each sample (A and B), and that the scattering between distinct unit cells is incoherent. For example, in liquid water, the observed coherence length is ≲15 Å (Skinner *et al.*, 2013 ▸), which is much smaller than the typical coherence volume of synchrotron X-rays. Then it is sufficient to consider the scattering from just one such volume, averaged over all thermally accessible atomic configurations; hereafter, we will implicitly assume all quantities are ensemble-averaged, and thus azimuthally isotropic.

Considering sample A [Fig. 1 ▸(*a*)], we can subdivide the total collection of atoms 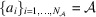 into the set of solute atoms 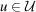 and the set of solvent atoms 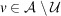 . From equation (3), the total distinct scattering intensity from this volume element of measurement A can be decomposed 

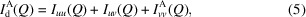

where the first term is summed over solute–solute pairs, the second is summed over solute–solvent pairs and the final term is summed over the solvent–solvent pairs (Dohn *et al.*, 2015 ▸, 2023 ▸); the superscript in the final term will prove necessary to distinguish it from an analogous term arising from the set of all atoms contained in an equivalent volume element of sample B, denoted by 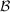 . Indeed, for sample B, all of the atoms can be classified into solvent atoms 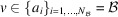 , hence the distinct scattering from measurement B is simply

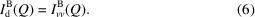

The total ‘differential’ distinct scattering intensity arising from the atoms in *V* is thus 

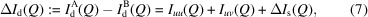

where 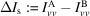 arises from the contrast in the solvent–solvent correlations between the two measurements.

As we are interested in the solvation structure of the solute, it is natural to normalize Δ*I*_d_ to the scattering power of the solute 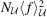 , in analogy to the standard formalism (the subscripts indicating that we consider only the set of solute atoms 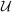 ). This choice is also congruent with previously reported differential solution scattering experiments (Yang *et al.*, 2016 ▸; Xie *et al.*, 2022 ▸). We thus define the ‘differential’ reduced total scattering structure function by 

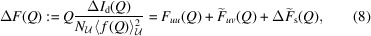

which follows from equation (4) if we use 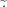 to denote that the corresponding term is normalized to the scattering power of the solute, rather than the scattering power of the atoms contributing to the interference function, as is usual [*c.f.* equation (4)].

The most immediate and obvious consequence of equation (8) is that 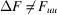 , that is, in the differential setting, the derived interference function is not identical to the standard one as though we had measured the solute alone. Although 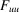 is the dominant term in the differential (see below), the remaining two terms make non-negligible contributions to the total scattering differential. The solute–solvent term, 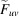 , provides direct structural information pertaining to the local ordering of solvent about the solute. The differential solvent–solvent contribution, 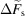 , might be called the ‘solvent-restructuring’ term, in that it originates from the contrast between the solvent–solvent atomic pair-correlations of the two measurements. However, as we shall now show, this term is complicated by excluded-volume effects, which must be accounted for before interpreting 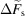 in terms of solvent reorganization.

### 2.3. The solvent excluded volume

Referring to the schematics in Fig. 1 ▸, we recognize that, provided the solution behaves approximately ideally (*i.e.* the solvent–solvent interactions are unperturbed by the presence of the solute), the liquid unit cell of measurement A will always contain fewer solvent atoms than an equivalent volume of measurement B owing to the finite volume of the solute. Hence, the differential solvent–solvent structure function, 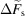 , must be corrected for this effect before interpreting how the solute affects the solvent structure. This is a necessary correction, because otherwise the solvent–solvent signals from the two samples are incommensurate, owing to the presence of excess solvent in sample B (or, equivalently, the absence of excluded solvent) (Soper, 1997 ▸).

Concretely, the surface of the van der Waals volume of the solute ( 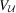 ) demarcates the minimal volume of space where solvent is expected to be excluded in sample A, *i.e.* it defines the ‘excluded volume’ (Soper, 1997 ▸). Of course, in actuality, if the solute particle is interacting strongly with the solvent, it will affect the short-range interactions present between solvent molecules in the solution A, leading to a genuine restructuring of the ‘bulk’ solvent. To assess the degree of this restructuring, 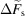 should be corrected for the finite volume of the solute particle. Letting *n*_B_ denote the macroscopic atomic number density of the solvent in B, the number of excess (or excluded) solvent atoms in the volume *V* of sample B is given by 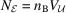 ; for each thermally accessible configuration of sample B, the excluded volume contains a ‘droplet’ of such atoms 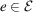 , which, on average, sum to 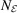 atoms. This leads to a natural partitioning of the total set of solvent atoms in 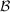 into 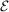 and its complement 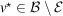 [Fig. 1 ▸(*b*)].

In analogy to the decomposition of equation (5), we can express the distinct scattering from measurement B as 

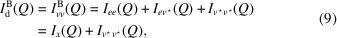

where we have grouped the terms 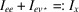 , given that both arise from correlations involving the excess or excluded solvent atoms. Note that total scattering experiments conducted in the HEXS regime typically do not collect data in the small-angle region (*i.e.* *Q*_min_ ≳ 1 Å^−1^) and are thus less sensitive to the nanoscopic morphology of the solvent excluded volume, as opposed to merely its size (2π/*Q*_min_ ≃ 10 Å). However, *a priori*, it is not clear at which precise value of *Q*_min_ the data become insensitive to the nanoscale details, hence, below we simply calculate *I_x_* directly from atomistic models, as detailed in the Section S3.2 of the supporting information (see Section S3.2.1 of the supporting information for a more detailed discussion of the nanoscopic sensitivity of the excluded-volume term).

Following equations (7) and (8), we can thus expand the solvent–solvent differential 

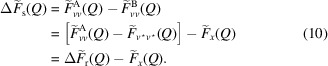

The physical meaning of the remaining differential, 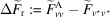 , can now be properly understood by ‘solvent restructuring’ in that it describes how the introduction of the solute changes the solvent structure ‘outside of the excluded-volume region’.

Another way to appreciate the consistency of this concept is to consider the asymptotic limits of each term in equation (10) as a function of the volume fraction of the solute, ν. As 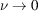 , the excluded volume vanishes, and both 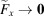 and 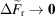 , implying 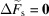 ; sample A becomes identical to sample B. As 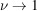 , there is no longer any solvent remaining in sample A, hence, tautologically, there can be no solvent reorganization ( 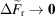 ); however, 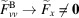 , and thus 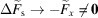 . In this limit, all of the solvent becomes ‘excluded’.

Although these are pure *gedanken* experiments, they serve to illustrate the necessity of separating out the excluded solvent structure function from the total differential solvent–solvent term before assessing the importance of solvent reorganization in a real experiment. At any finite ν, both 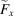 and 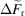 can be expected to contribute to the total differential signal, although for solutes that interact weakly with the solvent (*i.e.* ideal–dilute solutions), 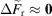 (see below).

### 2.4. Summary of the framework

To summarize, we are concerned with the structural information encoded in a differential total scattering experiment, 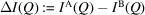 . In Section 2.2, we arrived at an exact formulation for the differential reduced total scattering structure function, Δ*F*(*Q*), equation (8). Though this is an exact result, we have further introduced the concept of the solvent excluded volume in order to decompose the differential solvent–solvent structure function, 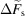 , into the sum of two contributions, one arising from the fact that the solute displaces a finite volume of solvent upon dissolution ( 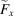 ), and one arising from genuine restructuring of the solvent upon perturbation by the solute ( 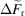 ).

Thus, combining equations (8) and (10), we arrive at a parameterization of the differential reduced structure function

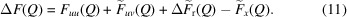

Since the notion of the solvent excluded volume is imposed *a priori* to decompose the pair-correlations from the pure solvent measurement B, equation (11) represents our proposed model for representing the information in the differential structure function. While we have motivated this decomposition physically, by reference to the finite volume of the solute in measurement A, the solvent excluded volume remains an imprecise, if useful, definition, depending as it does on a choice for computing the solute van der Waals volume.

The first three terms in equation (11) correspond to the solvation structure of the solute, understood with respect to the intra- and intermolecular interactions present in the solution, and its deviation from ideal-dilute behavior. The solute–solute structure function ( 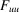 ) encodes the intramolecular structure of the solute in solution, averaged over the thermal ensemble. The solute–solvent structure function ( 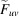 ) encodes the local ordering of solvent atoms about the solute, recovering the usual notion of the ‘solvation cage’ (Kim *et al.*, 2009 ▸; Ihee, 2009 ▸; Haldrup *et al.*, 2010 ▸). These terms are measured directly from the solution sample A. The solvent-restructuring structure function ( 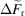 ) is defined by the contrast between measurements A and B, and in this sense can be considered an emergent function of the differential experiment. While 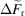 does not directly encode any two-body information regarding the solute atoms, it provides a concrete measure of the ideality of the solution, from a structural perspective (Martin & Shipman, 2023 ▸). By definition, in an ideal-dilute solution, the solvent–solvent interactions are unaffected by the presence of the solute and, hence, can be identified with the case that 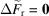 .

The excluded solvent structure function ( 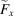 ), however, represents an essentially artifactual term arising from the differential approach, in that it does not encode any two-body information from the solution sample A. Although it is defined in reference to the solute structure, as a function of 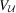 , the inversion of this mapping (*i.e.* obtaining 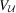 from 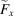 ) is likely to be better addressed via SAXS (*c.f.* Section 3.2.1 of the supporting information) (Jeffries *et al.*, 2021 ▸). As such, 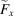 can be considered a ‘necessary evil’ of the differential solution HEXS experiment.

Finally, the ‘differential reduced PDF’ is naturally defined to be the Fourier sine transform of Δ*F*(*Q*) which, owing to the linearity of the transform, gives

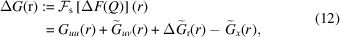

where, again, we use 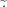 to remind us that the corresponding functions are transformed from renormalized structure functions. Equation (12) emphasizes the importance of a detailed theoretical understanding of the information content of the differential solution scattering experiment. If, in deriving a PDF from such an experiment, one assumes that subtracting a pure solvent reference from the raw solution scattering signal eliminates all solvent–solvent correlations, one might erroneously attribute features arising from 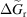 or 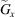 to the solute structure. Modeling an experimentally obtained PDF with 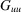 alone (Megyes *et al.*, 2003 ▸, 2005 ▸; Neuefeind *et al.*, 2004 ▸), or indeed using a ‘dummy atom’ approach to account for the excluded volume (Svergun *et al.*, 1995 ▸; Tiede *et al.*, 2004 ▸; Tiede *et al.*, 2009 ▸), may serve for an approximate description of the intramolecular structure of the solute. To achieve a quantitative description of the solvation structure, a more rigorous model is required (see below).

## 3. Numerical evaluation

To evaluate our model numerically, we simulated the differential solution scattering experiment using molecular dynamics (MD). By explicitly constructing two ensembles corresponding to samples A and B, we can perform an exact MD experiment [within the accuracy of the force field parameterization and the numerical precision in evaluating equation (8)] and analyze the results in terms of equation (11). As a test system, we considered aqueous solutions of tris(2,2′-bipyridine)ruthenium(II) dichloride ([Ru(bpy)_3_]Cl_2_), a classic transition metal complex whose solution structure has been the subject of numerous theoretical investigations (Moret *et al.*, 2009 ▸; Josefsson *et al.*, 2016 ▸; Abedi *et al.*, 2019 ▸). See the supporting information for complete details regarding the MD simulations and subsequent calculation of the coherent scattering intensities.

### 3.1. Construction

We first simulated sample A as a 15 m*M* aqueous solution of [Ru(bpy)_3_]Cl_2_ by solvating a single solute molecule in a box containing 3535 water molecules, and performing MD in the isothermal–isobaric (*NpT*) ensemble for 100 ns at 300 K and 1 atm (in five independent 20 ns trajectories). To properly simulate sample B, we must determine the (ensemble-averaged) number of excluded solvent atoms, 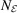 . A simple heuristic procedure is to estimate 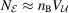 (see above), where *n*_B_ is the (experimental) atomic number density of the solvent (for water, *n*_B_ = 0.1 atoms Å^−3^). Using this heuristic, we find 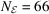 atoms, or 22 water molecules, hence sample B was simulated by performing MD on a box of 3557 water molecules for 20 ns in the *NpT* ensemble at 300 K and 1 atm.

To investigate the limits of the dilute solution phase for this system, we performed an additional solution simulation at 0.5 *M*, constructed from the pure solvent reference by replacing 22 water molecules for each copy of [Ru(bpy)_3_]Cl_2_ included in the simulation cell (32). We found that this system behaves identically up to 0.5 *M* (see the supporting information for further discussion), and restrict our discussion to the 15 m*M* simulation hereafter as this condition matches our experimental results below.

### 3.2. Evaluating the model

**Figure 2:**
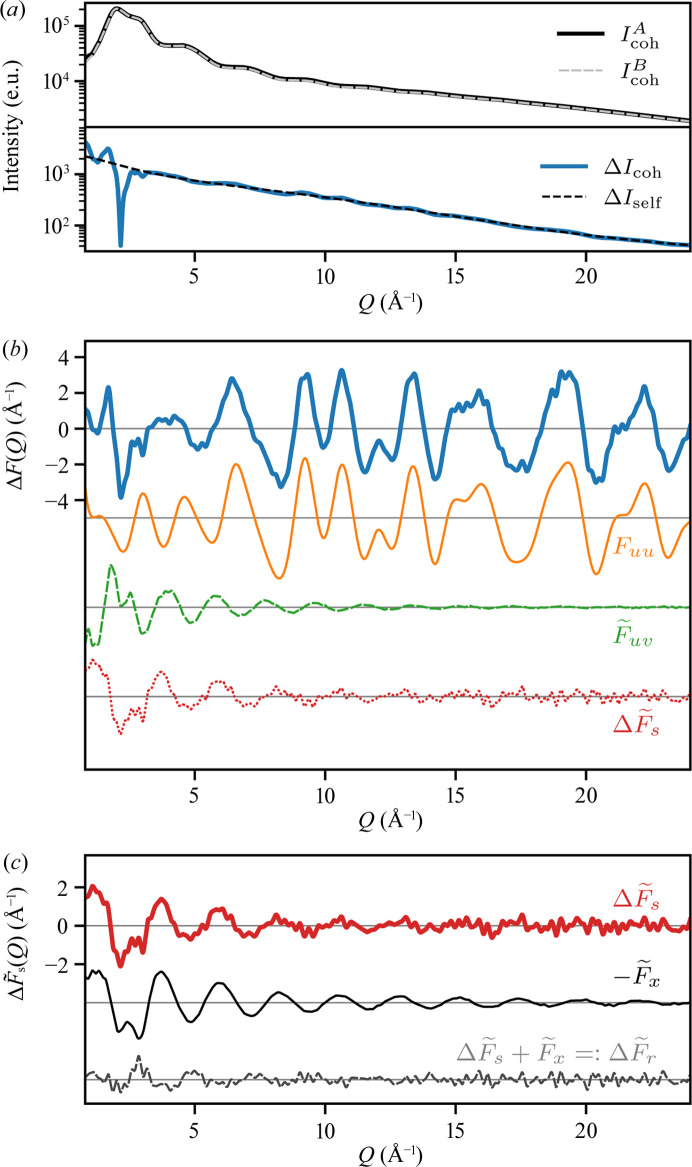
Simulation of the differential solution scattering experiment. (*a*) Top, total coherent scattering intensity from an MD simulation of a 15 m*M* aqueous solution of [Ru(bpy)_3_]Cl_2_ ( 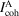 , solid black line) and a corresponding simulation of neat water ( 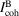 , dashed gray line); bottom, the total differential coherent scattering intensity (Δ*I*_coh_, solid blue line) along with the differential self-scattering (Δ*I*_self_, dashed black line). (*b*) The differential reduced structure function, Δ*F* (solid blue line), and its decomposition into 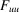 (solid orange line), 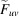 (dashed green line) and 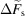 (dotted red line). (*c*) Further decomposition of 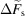 (solid red line) into 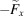 (solid black line) and 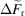 (dashed gray line).

As expected, the total coherent scattering intensity computed from the liquid unit cell of a 15 m*M* solution of [Ru(bpy)_3_]Cl_2_ is nearly indistinguishable, by eye, from that computed for an equivalent volume of neat water [Fig. 2 ▸(*a*), top]. The differential scattering intensity exhibits both a smoothly decreasing background, arising from the differential self-scattering, as well as the oscillatory features arising from the differential interference function (Δ*I*_d_), and is about 100 times weaker than the total coherent scattering intensities [Fig. 2 ▸(*a*), bottom]. Normalizing the interference function to the scattering power of [Ru(bpy)_3_]Cl_2_ and weighting the result by *Q* produces the differential reduced structure function Δ*F* defined in equation (8) [Fig. 2 ▸(*b*)].

Fig. 2 ▸(*b*) also shows the contributions to Δ*F* from each term in equation (8), illustrating that, while 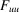 is in general large throughout reciprocal space (as a result of the rigid intramolecular structure of [Ru(bpy)_3_]^2+^), the remaining terms make non-negligible contributions at low- to moderate-*Q*. For example, the solute–solvent structure function ( 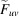 ) produces a strong peak at *Q* ≈ 1.7 Å^−1^, reminiscent of the ‘first sharp diffraction peak’ often observed in network-forming liquids and glasses (Elliott, 1995 ▸). The differential solvent–solvent structure function ( 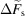 ) noticeably modulates the intensity throughout 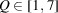 Å^−1^.

To further decompose 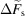 into the excluded solvent and solvent-restructuring terms, we performed an independent calculation of 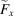 , and take 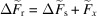 , by definition, following equation (10). To compute 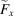 , we adopt the following strategy. For each particle of the solute (*i.e.* [Ru(bpy)_3_]^2+^ and two solvated Cl^−^ counterions), we use a representative molecular structure (*e.g.* for [Ru(bpy)_3_]^2+^, we used the ground-state structure optimized via DFT), and for each frame of simulation B we embed this structure into the center of the simulation. For a given frame, we find all solvent atoms that lie within one van der Waals radius of any atom of the solute particle, and label them as belonging to the excluded solvent droplet. In this fashion, all solvent atoms in all frames of the trajectory are labeled as belonging to the set 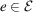 or the set 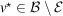 [*c.f.* Fig. 1 ▸(*b*)]. The total coherent scattering intensity calculated from simulation B is thus decomposed in accordance with equation (9). Note, therefore, that our evaluation of the solvent-restructuring term, 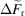 , is dependent on the precise algorithm used to perform this decomposition (*c.f.* Section S3.2 of the supporting information). However, the approach described above produces self-consistent results with our semi-empirical heuristic for 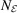 used in construction of the simulation, finding that 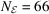 atoms in the ensemble-average.

As shown in Fig. 2 ▸(*c*), 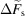 is dominated by the excluded solvent structure function ( 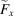 ). The amplitude of the solvent-restructuring structure function ( 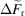 ) damps nearly to zero by *Q* ≈ 6 Å^−1^, while the ratio 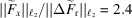 over 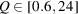 Å^−1^. This numerical result indicates that, at least within the accuracy of our simulation, and our algorithm for computing 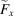 , [Ru(bpy)_3_]Cl_2_ does not significantly restructure water. Without a more detailed investigation, beyond our scope here, we also caution that the sensitivity of 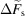 to restructuring (*i.e.* the region where 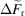 is different from zero) is likely to be localized to the low-*Q* region, which is typically truncated in data collection optimized for HEXS-based PDF studies (*c.f.* Section S3.2.1 of the supporting information).

**Figure 3:**
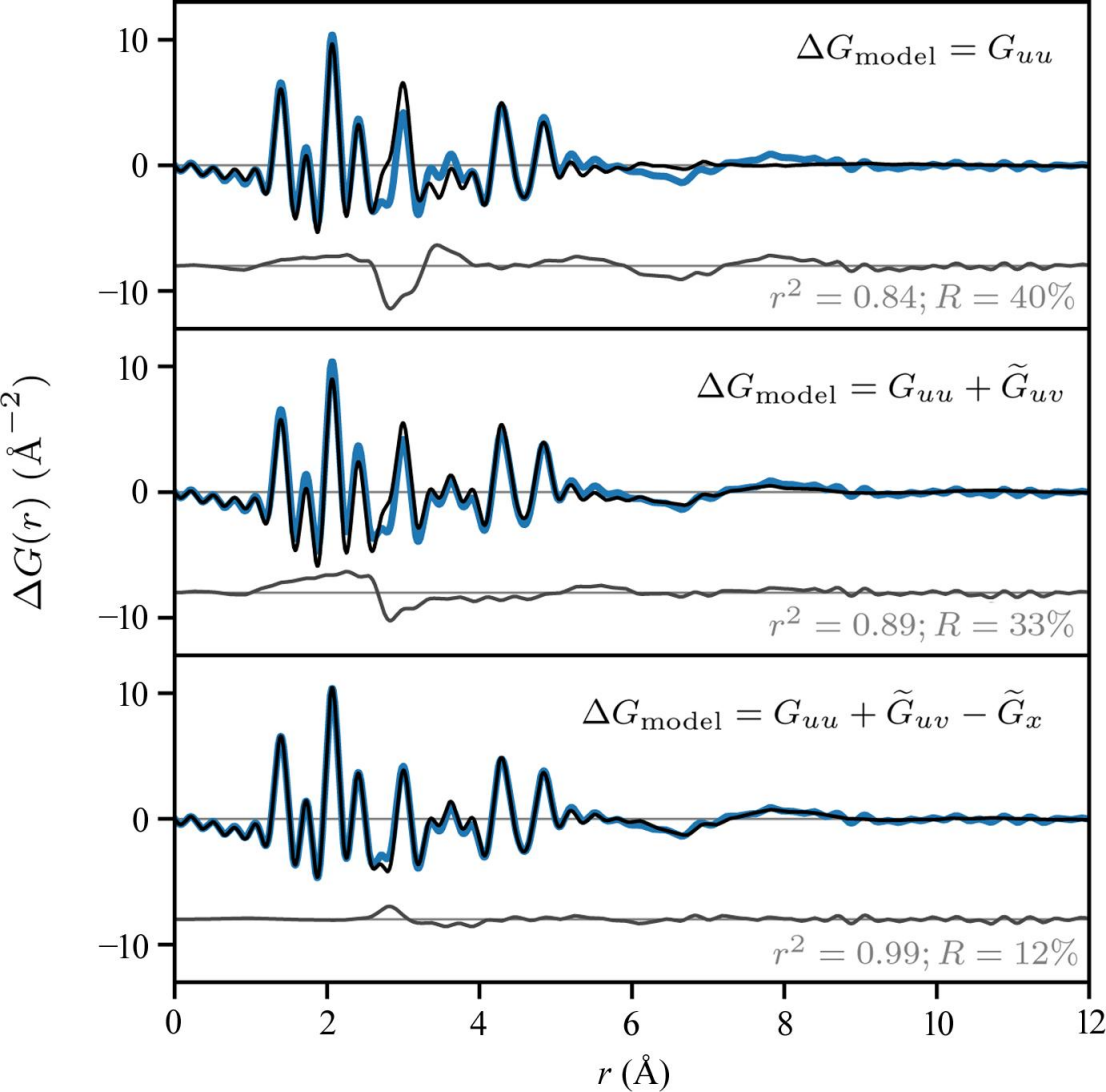
Various approximations to Δ*G*. Each plot compares the exact Δ*G* (solid blue line) from the simulated experiment with various models (Δ*G*_model_, solid black line), as indicated, of increasing complexity. The residuals are shown below in gray. Data are the Fourier transforms of those in Fig. 2 ▸(*b*) using 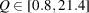 Å^−1^.

Now we wish to understand the relative importance of each term summing to our complete model. For this, we require a statistical measure of the contribution of each of these terms to approximate the total result of equation (11). As our focus is on developing a PDF formalism, we will perform this analysis on the real space analogs [equation (12)]. Considering Δ*G* as ground truth, one measure of the explanatory power of a model is given by the coefficient of determination (*r* ^2^) which ranges from 0 (baseline model) to 1 (perfect linear model); alternatively, we can judge the goodness-of-fit of each model via the standard crystallographic *R* factor. Table 1 ▸ lists both metrics for a series of models given by sequential addition of each term in equation (12); these same data are presented graphically in Fig. 3 ▸.

From these results, it is clear that 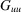 dominates the total PDF, as expected (*r* ^2^ = 0.84; *R* = 40%). However, to achieve near quantitative accuracy, it is necessary to consider both 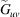 as well as 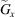 (*r* ^2^ = 0.99; *R* = 12%). Indeed, in terms of accuracy, the excluded solvent structure matters more than the solute–solvent structure, despite its ‘artifactual’ nature. In this system, the solvent restructuring appears nearly negligible (see above); the *R* factor for a model neglecting this term (12%) is already about a factor of 2 to 3 times lower than typically achieved in modeling PDFs of molecular systems (20–30%) (Terban & Billinge, 2022 ▸). Nevertheless, in general the importance of solvent restructuring should be assessed on a case-by-case basis, by independent thermodynamic measures of solution ideality, for example.

## 4. Experimental validation

To validate our framework against experimental data, we performed total scattering measurements at beamline 11-ID-B of the Advanced Photon Source. We collected data on a 15 m*M* aqueous solution of [Ru(bpy)_3_]Cl_2_ and an equivalent sample of neat water in the range 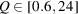 Å^−1^, and reduced these data to Δ*F*. In practice, this requires access to flux-normalized total scattering data, which are often not available with the required precision at PDF-focused beamlines such as 11-ID-B. Typically, the solution to this problem would be to obtain absolutely normalized total scattering data during reduction, using the high-*Q* behavior of the raw data to compute normalization terms, as it is dominated by the structure-independent Compton scattering.

However, since this requires high-quality data collected out to high-*Q* and accurate calculation of the Compton backgrounds (which in turn demands rigorous precision in the preparation of dilute solutions), we detail an alternative approach for data reduction that automates this task in the supporting information. Briefly, from the derivation of the theoretical framework above, it is straightforward to perform data normalization and subtraction simultaneously, utilizing the standard information required for reduction of total scattering data to a PDF, but incorporating priors regarding the solvent excluded volume and the ideality of the solution to render the reduction a convex program. See Section S4 of the supporting information for full details on the data reduction algorithm.

**Figure 4:**
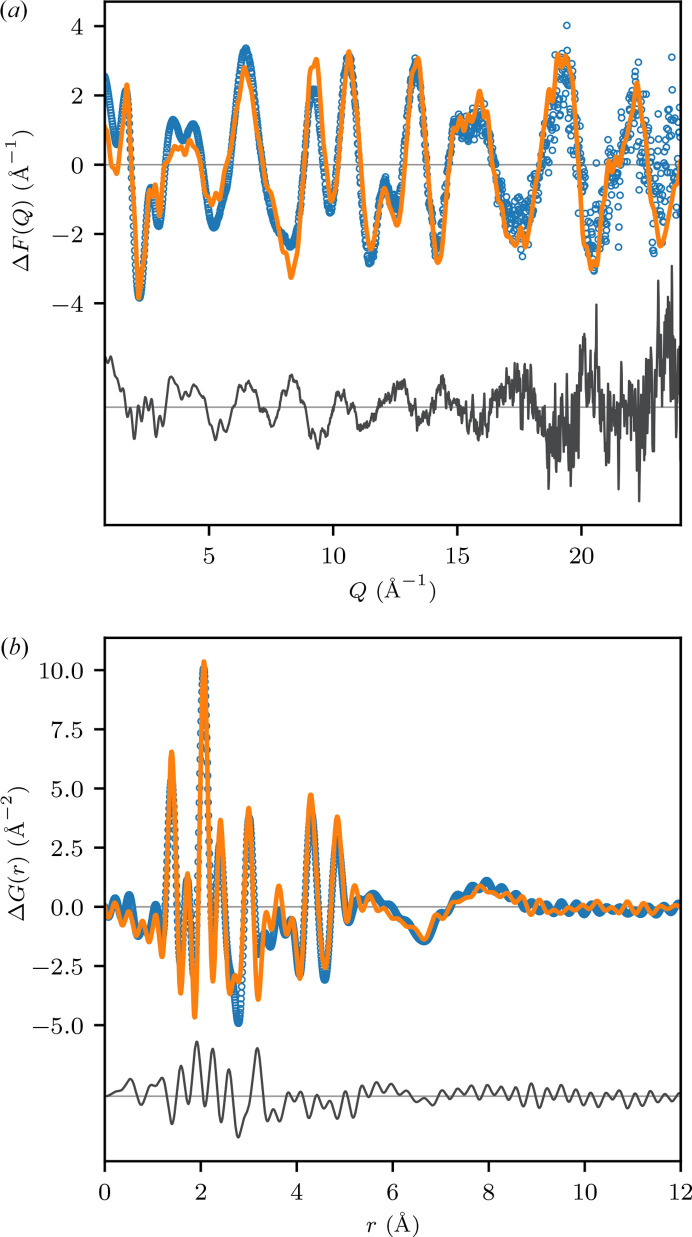
Comparison of the experimental solution scattering data with MD simulation. (*a*) Comparison of the experimental Δ*F* (open blue circles) derived from a 15 m*M* aqueous solution of [Ru(bpy)_3_]Cl_2_ with calculation (solid orange line); the residual is shown below in gray. (*b*) Data from (*a*) Fourier transformed to real space using 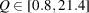 Å^−1^.

The experimental Δ*F* is shown in Fig. 4 ▸(*a*), alongside our MD calculation. Qualitatively, the agreement is excellent: every feature observed in the experiment is reproduced by the calculation. The residual is dominated by modulations that persist throughout reciprocal space, indicating that the largest source of error in our simulation lies in the intramolecular solute–solute structure. This is borne out in real-space analysis [Fig. 4 ▸(*b*)], where the majority of the residual arises from small errors in the peak profiles below *r* ≃ 5 Å, corresponding to the sharp, intramolecular features attributable to [Ru(bpy)_3_]^2+^. Significantly, at *r* ≳ 5 Å, the residual drops to the level of the Fourier truncation ripples in the experimental data. As the features at long-*r* arise from the subtle combination of the solute–solvent, excluded solvent and solvent-restructuring terms (Fig. 3 ▸), the quantitative agreement in this region of real space indicates that our simulation accurately captures these structural features.

Given the difficulty in constructing accurate molecular mechanics force fields for coordination complexes (Deeth, 2010 ▸), the errors in our simulated 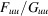 are unsurprising. Nevertheless, the overall level of agreement is remarkable considering that no attempt has been made here to optimize the simulation against the experimental data. Indeed, the most significant conclusion to be drawn from these results is that our framework provides a realistic and accurate parameterization of the differential solution scattering experiment. Such a parameterization is a necessary prerequisite for solving the inverse modeling problem (*i.e.* solving the solution structure from experimental scattering data).

To solve this inverse problem, one could, for example, use the experimental PDF to drive the optimization of the force field (Hsu *et al.*, 2020 ▸). Alternatively, if classical force fields are unsuitable (a likely scenario for transition metal complexes), one could use quantum-mechanically derived normal modes to parameterize the internal degrees of freedom of the solute (*i.e.* 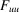 ), and combine this with a coarser-grained MD parameterization for the remaining terms (Leshchev *et al.*, 2023 ▸). Discussion of the structural features in the experimental scattering data of [Ru(bpy)_3_]Cl_2_, in terms of a refined theoretical model, is beyond the scope of this work, and will be presented in a subsequent publication. Here, we simply note that both our simulated and experimental results are in broad agreement with prior theoretical studies of the aqueous solvation structure of [Ru(bpy)_3_]Cl_2_, in particular in the presence of two resolved solvation shells with peak maxima near 5.5 and 8.0 Å [Fig. 4 ▸(*b*)] (Moret *et al.*, 2009 ▸; Josefsson *et al.*, 2016 ▸; Abedi *et al.*, 2019 ▸).

## 5. Conclusions

Although solvation can be a critical determinant of chemistry in the condensed phases, few experimental techniques are capable of determining the structure of molecules in solution with sub-atomic resolution. In principle, total X-ray scattering measurements yield such information, but are complicated by the necessity of a differential analysis to extract the relevant scattering data against a large experimental background. In order to fully understand the information content of such experiments, we have developed a theoretical framework for differential solution scattering, provided by equations (11) and (12). This framework was evaluated with the aid of numerical simulations and validated by comparison to experiment.

Our results lay the groundwork for developing quantitative simulations of solvation structure, as measured by total X-ray scattering. Efforts directed at evaluating quantitative simulation methodologies are ongoing at Argonne. Ultimately, one can envision that solution scattering measurements will provide insights into atomic structure with accuracy and precision approaching crystallography, but instead of reporting on the average (macroscopic) structure of a molecule in a crystal, elucidating the local (nanoscopic) structure of a molecule in complex condensed phases.

While our focus has been on solvation structure, the application of total scattering measurements to complex, multiphasic systems is increasingly popular (Jensen *et al.*, 2015 ▸; Roelsgaard *et al.*, 2019 ▸; He *et al.*, 2020 ▸; Hoffman *et al.*, 2024 ▸). In these settings, challenges similar to those of the solution scattering experiment arise (*e.g.* a large experimental background from a bulk substrate onto which a thin film of interest is deposited). Though not immediately applicable, we believe that the methodology described here should provide a useful framework for understanding the data acquired in any total scattering experiment where a differential analysis proves necessary.

## 6. Related literature

The following references are cited in the supporting information: Frisch *et al.* (2016 ▸); Staroverov *et al.* (2003 ▸); Weigend & Ahlrichs (2005 ▸); Singh & Kollman (1984 ▸); Besler *et al.* (1990 ▸); Rappe *et al.* (1992 ▸); Li & Merz (2016 ▸); Case *et al.* (2021 ▸); Izadi *et al.* (2014 ▸); Sengupta *et al.* (2021 ▸); Eastman *et al.* (2017 ▸); Warren (1990 ▸); Neder & Proffen (2020 ▸); Juhás *et al.* (2015 ▸); Waasmaier & Kirfel (1995 ▸); McGibbon *et al.* (2015 ▸); Thijsse (1984 ▸); Cromer & Mann (1967 ▸); Toby & Von Dreele (2013 ▸); Peterson *et al.* (2021 ▸); Qiu *et al.* (2004 ▸); Weng *et al.* (2023 ▸); Juhás *et al.* (2013 ▸). 

## Supplementary Material

Force field. DOI: 10.1107/S2052252524003282/ro5040sup1.txt

Supporting information, equations and figures. DOI: 10.1107/S2052252524003282/ro5040sup2.pdf

## Figures and Tables

**Table 1:** Statistical decomposition of the framework

Model	*r* ^2^	*R* factor (%)
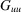	0.84	40
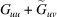	0.89	33
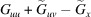	0.99	12
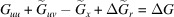	1.00	0.0
