# Supplementary material for: Toward a quantitative description of solvation structure: a framework for differential solution scattering measurements
Source: IUCrJ. 2024 May 1;11(Pt 3):423–33. doi: 10.1107/S2052252524003282 (PMC11067739; doi:10.1107/S2052252524003282)
Supplement: Supplementary file 2 [file m-11-00423-sup2.pdf]

IUCrJ

Volume 11 (2024)

Supporting information for article:

Toward a quantitative description of solvation structure: an *Ansatz* for differential solution scattering measurements

Niklas B. Thompson, Karen L. Mulfort and David M. Tiede

Supporting Information for

Toward a Quantitative Description of Solvation Structure: An *Ansatz* for Differential Solution Scattering Measurements

Niklas B. Thompson,* Karen L. Mulfort and David M. Tiede*

Division of Chemical Sciences and Engineering, Argonne National Laboratory, 9700 S. Cass Ave., Lemont, Illinois 60439, United States.

*Correspondence e-mail: niklas@anl.gov, tiede@anl.gov

Contents

1 Experimental Methods **2**
1.1 General Considerations 2
1.2 Total Scattering Measurements 2

2 Computational Methods **3**
2.1 Density functional theory calculations 3
2.2 Force field parameterization 3
2.3 Molecular dynamics simulations 3
2.4 Simulating total scattering 4

3 Numerical Results **6**
3.1 Construction of simulation cells 6
3.2 Calculating the excluded solvent structure 7
3.2.1 Sensitivity of the excluded volume to nanoscopic morphology 8
3.3 Evaluating concentration dependence 10

4 Experimental data reduction **11**

Supplementary References **16**

1 Experimental Methods

1.1 General Considerations

All samples were prepared under ambient conditions, using ultra-pure (Milli-Q) water. $[Ru(bpy)_3]Cl_2$ (bpy = 2,2'-bipyridine) was obtained from commercial sources and used without further purification.

1.2 Total Scattering Measurements

High energy X-ray scattering measurements were performed at beamline 11-ID-B of the Advanced Photon Source at Argonne National Laboratory. All measurements were performed with an unfocused (0.5 × 0.5 mm) incident X-ray beam of energy 58.6 keV (λ = 0.2115 Å), and an amorphous silicon detector (Perkin Elmer XRD1621). Samples were loaded into polyimide capillaries (Cole-Parmer; ID = 1.275 mm, OD = 1.367 mm), sealed with epoxy, and mounted in a plastic sample holder. Images were collected by integrating 0.4 s exposures (well-within the dynamic range) for 5 min before allowing the detector to relax for an additional 5 min; replicate 5 min integrations were performed until good statistics were achieved to at least $Q \approx 20$ Å^{-1}. All measurements were performed with a nominal sample-to-detector distance of 180 mm, which was calibrated using a CeO_2 reference sample. After subtraction of dark counts, a flat field correction was applied to each raw image using the program QXRD. See §4 for further details regarding the reduction of the experimental data.

In all, we performed duplicate measurements on independently-prepared samples of aqueous $[Ru(bpy)_3]Cl_2$ (ca. 15 mM) and neat water, totaling 50–60 min of total integration time for each duplicate sample (or, ca. 2 h total time for the solution measurement and the solvent measurement, after subsequent averaging).

2 Computational Methods

2.1 Density functional theory calculations

All DFT calculations were performed using Gaussian 16 [1]. Gas-phase geometry optimizations and frequency calculations were carried out with the hybrid meta-GGA exchange correlation functional TPSSh [2] along with the def2-TZVP basis set on all atoms [3]. Following geometry optimization, molecular electrostatic potential maps were computed at the same level of theory according to the Merz-Kollman-Singh (MKS) scheme [4, 5], using the standard MKS atomic radii for main group elements and those given by Rappe, *et al.* for transition metals [6].

2.2 Force field parameterization

Force fields for transition metal complexes were parameterized with MCPB.py using the Seminario method for bonded parameters and the RESP method for charges [7], based on the DFT calculations described above. Bonded parameters involving ligand atoms not directly attached to the metal center were parameterized using version 2 of the General Amber Force Field (GAFF2) distributed with AmberTools21 [8]. The rigid 4-point OPC force field was used to model solvent water and counterions [9, 10]. All force fields are provided in the form of OpenMM-compatible *.xml files as supplementary information.

2.3 Molecular dynamics simulations

Molecular dynamics (MD) simulations were performed using OpenMM [11]. All simulation cells were cubic (*ca.* 48 Å edge length), and treated using periodic boundary conditions in the minimum image convention. Simulations used a `LangevinMiddleIntegrator` with a time step of 2 fs and a friction coefficient of 1/ps. Lennard-Jones forces were smoothly damped to zero starting at a distance of 12 Å to the long-range cutoff at 15 Å. Long-range nonbonded interactions were treated with the Particle Mesh Ewald method for both Coulomb and Lennard-Jones forces using the same cutoff distance of 15 Å. All bonds were constrained during simulation and water molecules were treated as fully rigid. Simulations were initialized at 1 K, minimized, and heated linearly to 300 K in 500 ps in an NVT ensemble. Following heating, simulations were equilibrated at 300 K in an NpT ensemble at 1 atm for an additional 500 ps; pressure coupling was achieved with a `MonteCarloBarostat` with a coupling frequency of 25 time steps. Finally, production runs were performed for 20 ns in the NpT ensemble, sampling snapshots every 10 ps, for a total of 2000 frames. In the case that additional statistics were required, multiple independent 20 ns trajectories were

computed, and the results of subsequent calculations averaged.

2.4 Simulating total scattering

To simulate the coherent scattering from atomic coordinates given by an MD trajectory, we follow the approach of Dohn, *et al.* [12] (*c.f.* Warren, pp. 135–142 [13]). This method, which is mathematically equivalent to evaluating the Debye scattering equation, takes advantage of efficient algorithms to compute radial pair distribution functions (RDFs) from MD data, and encodes thermal broadening effects in a non-parametric fashion directly from the simulation dynamics. Moreover, it avoids invocation of the Morningstar-Warren approximation often used by other RDF-based approaches to calculating coherent scattering intensities [14].

Briefly, if we classify all atoms $\{a_i\}_{i=1,\ldots,N} = \mathcal{A}$ into types $\alpha, \beta, \ldots$ such that $\sum_\alpha N_\alpha = N$ (where N_α is the number of atoms of type α), and each type α is composed of a single element,* and we let $\{f_k\}_{k=1,\ldots,M}$ index the snapshots ('frames') defining the trajectory, then the coherent scattering intensity for a given frame of the trajectory can be computed according to,

$$
\begin{aligned}
I_{\mathrm{coh},k}(Q) = &\sum_{\alpha\in\mathcal{A}} N_\alpha f_\alpha(Q)^2 + \\
&\sum_{\alpha\in\mathcal{A}}\sum_{\beta\in\mathcal{A}} f_\alpha(Q) f_\beta(Q) \frac{N_\alpha\left(N_\beta - \delta_{\alpha\beta}\right)}{V_k} \int_0^\infty 4\pi r^2 \left(g_{\alpha\beta,k}(r) - g_{0,\alpha\beta}\right) \frac{\sin Qr}{Qr} \mathrm{d}r
\end{aligned}
\tag{S2.1}
$$

where $\delta_{\alpha\beta}$ is the Kronecker delta function, V_k is the volume of the simulation cell in frame f_k (hence, the prefactor to the integral is simply the product of the atomic number densities of types α and β), $g_{\alpha\beta,k}(r)$ is the pair radial distribution function (RDF) for atom types α and β computed from frame f_k, and $g_{0,\alpha\beta}$ is the (theoretical) limit of the RDF as $r \to \infty$ ($g_{0,\alpha\beta} = 1$ for collections of freely-diffusing atoms, while $g_{0,\alpha\beta} = 0$ for collections of atoms bound in a single molecule). The first term in the sum provides the self-scattering while the second provides the distinct scattering. Implicit in the double summation above is that when $\alpha = \beta$, only distinct atom pairs are selected in the computation of the RDF. Note that the distinct scattering term of equation (S2.1) naturally decomposes the total distinct scattering from the collection of atoms into a set of partial terms, $I_{\alpha\beta}(Q)$, such that $I_d(Q) = \sum_{\alpha,\beta} I_{\alpha\beta}(Q)$. If there are Γ atom types, then in general there are Γ^2 partials. However since $g_{\alpha\beta}(r) = g_{\beta\alpha}(r)$ it is sufficient to calculate $\frac{\Gamma(\Gamma-1)}{2}$ of the "cross-terms" when $\alpha \neq \beta$ and multiply them by a factor of 2.

*There may be distinct atom types of the same element, *e.g.*, an H atom of a water molecule will be treated as a different atom type than an H atom of the solute

Orientational averaging, as appropriate for isotropic systems, is achieved by computing the frame-averaged quantity (equivalent to the ensemble average assuming ergodicity),

$$I_{\text{coh}}(Q) = \langle I_{\text{coh},k}(Q) \rangle_k = \frac{1}{M} \sum_{k=1}^{M} I_{\text{coh},k}(Q) \tag{S2.2}$$

For trajectories computed in the NVT ensemble, Eqn. S2.2 reduces to a form analogous to Eqn. S2.1 where $V_k = V$ and the frame (ensemble) averaged RDF, $g_{\alpha\beta}(r) = \langle g_{\alpha\beta,k}(r) \rangle_k$, replaces $g_{\alpha\beta,k}(r)$; in this case, $g_{\alpha\beta}(r)$ can be computed directly from the simulation data to achieve orientational averaging. For NpT trajectories, where V is not conserved, the coherent scattering must be computed frame-wise according to Eqn. (S2.1), and subsequently averaged via Eqn. (S2.2). It is also important to note that the integral in Eqn. (S2.1) is truncated at a finite maximum radius R, typically taken as half of the simulation cell side length. Finite-size effects arising from this truncation have recently been investigated in detail [15]; however, as these typically result in errors at $Q < 1$ Å, we will not treat this topic further. We have implemented Eqns. (S2.1) and (S2.2) in Python. Atomic form factors were taken from the Python package Diffpy [16] (which themselves adopt the Waasmaier–Kirfel model [17]), while RDFs were computed using the Python package MDTraj [18]. Pending a full release as a package, a development version of the code used in this work is available online at: https://github.com/niklasbt/mdxcs_dev.

For a given collection of atoms, $\{a_i\}_{i=1,\dots,N}$, the average incoherent (Compton) scattering contribution *per atom* can be expressed,

$$\langle C(Q) \rangle = \frac{R^a}{N} \sum_{i=1}^{N} C_i(Q) \tag{S2.3}$$

where $C_i(Q)$ is an expression depending only on the atomic structure of atom i, and R^a is the so-called Breit-Dirac recoil factor (we assume $a = 2$ [19]). Hence, the incoherent contribution to the total scattered intensity is given by,

$$I_{\text{inc}}(Q) = N \langle C(Q) \rangle \tag{S2.4}$$

Eqn. (S2.4) was evaluated using Thijsse's empirical fitting [20] of the calculations of Cromer and Mann [21].

3 Numerical Results

3.1 Construction of simulation cells

As described in the main text, we simulated a dilute solution of $[Ru(bpy)_3]Cl_2$ by solvating a single solute molecule in a box containing 3535 water molecules. Given the final average volume of the trajectory (107 nm^3), this corresponds to a 15.5 mM solution. To estimate the ensemble-averaged size of the excluded solvent droplet ($N_{\mathcal{E}}$) for this solute, we require an estimate for the van der Waals volume, $V_{\mathcal{U}}$, of $[Ru(bpy)_3]Cl_2$. To do so, we must fix a set of atomic van der Waals radii, r_{vdW}; while there is not a unique choice of r_{vdW}, here we choose perhaps the most "natural" measure for the size of the atoms in our simulation, *i.e.*,

$$r_{\mathrm{vdW}} = \frac{\sigma}{2^{5/6}} \approx \frac{\sigma}{1.78} \tag{S3.5}$$

where σ is the Lennard-Jones parameter for the atom in our force field. That is, we take r_{vdW} to be one-half of the internuclear separation which minimizes the Lennard-Jones potential for the homodimeric system ($r_{\mathrm{min}} = 2^{1/6}\sigma$).

With this choice of r_{vdW} set, we can estimate $V_{\mathcal{U}}$ by enclosing a representative molecular structure of the solute particle into a three-dimensional box, choosing a particular voxelization of the box, and then simply summing the volumes of those voxels ($\sum \Delta V$) which intersect with any sphere of radius r_{vdW} centered about any atom of the solute. In the limit of the voxel dimension passing to the infinitesimal volume element ($\Delta V \to \mathrm{d}V$), this sum should approach $V_{\mathcal{U}}$. Concretely, to obviate over-counting voxels, we compute the function $f : \{V_i\} \to \{0, 1\}$ that maps the value of a voxel (V_i) to 0 if it does not intersect with any of the solute atoms, and 1 if it does; the total volume of the particle is then approximated by $V_{\mathrm{particle}} = \Delta V \sum_i f(V_i)$. We chose a voxelization that reproduces the expected volume for a single-atom particle with high precision; specifically, $\Delta V = 1 \times 10^{-4}$ Å^3 which reproduces the exact volume for a single chloride ion ($\frac{4}{3}\pi r_{\mathrm{vdW}}^3 = 55.1$ Å^3 for $r_{\mathrm{vdW}} = 2.36$ Å) within 3% error (56.8 Å^3). This is sufficiently accurate for our purposes, as we are restricted to whole numbers of atoms in constructing simulations.

In the case of $[Ru(bpy)_3]Cl_2$, where we expect dissolution to produce three solvated particles (*i.e.*, $[Ru(bpy)_3]^{2+}$ and two Cl^- counterions), this procedure needs to be performed on each particle separately. With $V_{\mathcal{U}}$ in hand, we can compute $N_{\mathcal{E}} = n_B \times V_{\mathcal{U}}$, where $n_B = 0.1$ atoms Å^{-3} is the experimental atomic number density for water. For $[Ru(bpy)_3]Cl_2$, we find that $N_{\mathcal{E}} = 65.9$, or, rounding to the nearest whole molecule, 22 water molecules. Thus, we simulated the pure solvent reference system by a cell containing 3557 water molecules. For solutes with many nearly-degenerate conformers, this procedure should be repeated by

considering the complete set of thermally-populated conformers. Thermally-equilibrated simulation cells are provided in the format of OpenMM-compatible *.pdb files as supplementary information.

3.2 Calculating the excluded solvent structure

To compute the excluded solvent structure function, we adopt a strategy similar to computing the van der Waals volume described above. That is, we consider each frame of the solvent reference trajectory (B), and place an *image* of a representative structure of the solute particle into the center of the pure solvent simulation cell. That is, we superimpose a representative molecular structure of the solute particle within the frame; here, we use the DFT-optimized structure of $[\mathrm{Ru(bpy)_3}]^{2+}$, although for less rigid solute particles, it may prove necessary to consider an ensemble of structures representing the thermal population of conformers. We then compute the pairwise distances between all solvent atoms and all atoms of the solute, and label all those which lie within one van der Waals radius of any solute atom as belonging to the excluded solvent droplet in that given frame. In this fashion, we partition each atom of the solvent, in each given frame, as belonging to either the excluded solvent droplet ($e \in \mathcal{E}$), or its complement ($v^\star \in \mathcal{B} \setminus \mathcal{E}$). With the atoms so-partitioned, it is straightforward to evaluate $I_{ee}(Q)$ and $I_{ev^\star}(Q)$ as described above.

More precisely, we evaluate the distinct scattering term of equation (S2.1), further decomposing the set of all elemental solvent atom types into sub-classes, according to whether the atom is labeled $e \in \mathcal{E}_k$ (the excluded solvent droplet in frame f_k) or $v^\star \in \mathcal{B} \setminus \mathcal{E}_k$. We can then construct,

$$I_{ee}(Q) = \left\langle \sum_{\alpha,\beta \in \mathcal{E}_k} I_{\alpha\beta} \right\rangle_k$$
$$\left\langle \sum_{\alpha,\beta \in \mathcal{E}_k} f_\alpha(Q) f_\beta(Q) \frac{N_\alpha \left(N_\beta - \delta_{\alpha\beta}\right)}{V_k} \int_0^\infty 4\pi r^2 \left(g_{\alpha\beta,k}(r) - g_{0,\alpha\beta}\right) \frac{\sin Qr}{Qr} \mathrm{d}r \right\rangle_k \tag{S3.6}$$

$$I_{ev^\star}(Q) = \left\langle \sum_{\alpha \in \mathcal{E}_k, \beta \in \mathcal{B} \setminus \mathcal{E}_k} I_{\alpha\beta} \right\rangle_k$$
$$\left\langle \sum_{\alpha \in \mathcal{E}_k, \beta \in \mathcal{B} \setminus \mathcal{E}_k} f_\alpha(Q) f_\beta(Q) \frac{N_\alpha \left(N_\beta - \delta_{\alpha\beta}\right)}{V_k} \int_0^\infty 4\pi r^2 \left(g_{\alpha\beta,k}(r) - g_{0,\alpha\beta}\right) \frac{\sin Qr}{Qr} \mathrm{d}r \right\rangle_k \tag{S3.7}$$

For the case of water as the solvent, there are thus four atom types, in total, i.e., H and O atoms in $\mathcal{E}_k$, and $\mathrm{H}^\star$ and $\mathrm{O}^\star$ atoms in $\mathcal{B} \setminus \mathcal{E}_k$. In this case, the sum in equation (S3.6) contains three partials (stemming from H–H, H–O, and O–O pairs *within* the excluded

volume droplet), while the sum in equation (S3.7) contains four partials (H–H*/H–O* and O–H*/O–O* pairs *across* the excluded volume interface). By definition, we arrive at our excluded solvent reduced structure function,

$$\widetilde{F}_x(Q) = \frac{Q}{N_{\mathcal{U}} \langle f(Q)\rangle_{\mathcal{U}}} \left[I_{ee}(Q) + I_{ev^\star}(Q)\right] \tag{S3.8}$$

keeping in mind that this quantity should be summed over all independent solute particles.

Finally, for completeness, the forward-simulation of the excluded solvent structure function allows us to compute the solvent restructuring structure function, again by definition,

$$\begin{aligned} \Delta\widetilde{F}_r(Q) &= \Delta\widetilde{F}_s(Q) + \widetilde{F}_x(Q) \\ &= \frac{Q}{N_{\mathcal{U}} \langle f(Q)\rangle_{\mathcal{U}}} \left[I^A_{vv}(Q) - I^B_{vv}(Q) + I_{ee}(Q) + I_{ev^\star}(Q)\right] \end{aligned} \tag{S3.9}$$

Equivalently, we could directly evaluate $I_{v^\star v^\star}(Q)$ following the scheme above, and compute $\Delta\widetilde{F}_r(Q) = Q/N_{\mathcal{U}} \langle f(Q)\rangle_{\mathcal{U}} \left[I^A_{vv}(Q) - I_{v^\star v^\star}(Q)\right]$.

3.2.1 Sensitivity of the excluded volume to nanoscopic morphology

Acknowledging that; (i) at high momentum transfers (high angles), the coherent scattering contribution to the total scattering signal is most sensitive to atomic-scale pair correlations; and that (ii), in practice, experimental HEXS studies typically report $Q_{\text{min}} \approx 0.5$ to 1 Å^{-1}, and thus include some amount of small-angle information; it is unclear, a priori, how sensitive $\widetilde{F}_x(Q)$ is to the nanoscopic morphology of the excluded volume particle. That is, it might be argued that the precise form of $\widetilde{F}_x(Q)$ should only be sensitive to the *volume* of the particle (a 1-dimensional quantity), and not its *morphology* (a 3-dimensional quantity), given the local character of the measurement. If that were the case, one could simply model

$$I_x(Q) \approx \nu I^B_{\text{d}}(Q) \tag{S3.10}$$

where ν is some scaling factor (intuitively, the volume fraction of the solute particle).

Although a complete evaluation of this approximation will relegated to future work, we have investigated its scope within our model system, viz. $[Ru(bpy)_3]^{2+}$. We have considered particles of approximately identical volumes, but distinct morphologies: (i) a simple sphere of radius $r = 5$ Å ($N_{\mathcal{E}} = 53$ atoms), (ii) a linear chain of four pseudo-atoms with $r_{\text{vdW}} = 3.75$ Å spaced 2.5 Å apart ($N_{\mathcal{E}} = 55$ atoms), (iii) a cubic array of eight pseudo-atoms with edge length 2.5 Å and with $r_{\text{vdW}} = 2.5$ Å ($N_{\mathcal{E}} = 54$ atoms), and (iv) $[Ru(bpy)_3]^{2+}$ itself, as described above ($N_{\mathcal{E}} = 54$ atoms, without considering the counterions). Using the

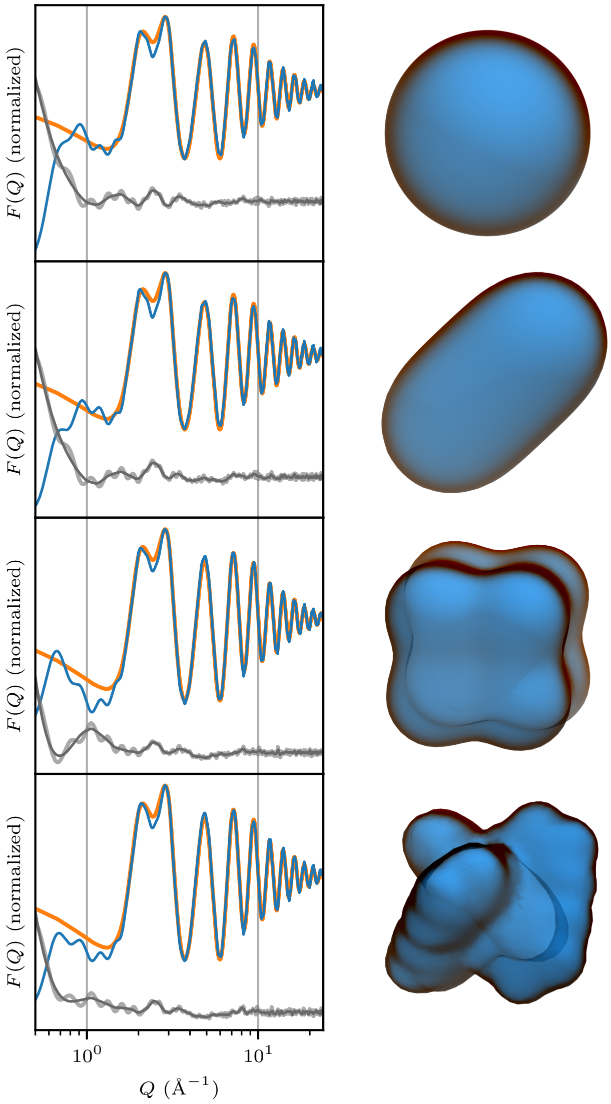

Figure S1 : Comparison of the normalized total scattering reduced structure function, $F(Q)$, computed for bulk water (thick orange line) with the excluded solvent structure function computed with different particle morphologies (thin blue line). The offset gray lines show the residual (light gray) along with a smoothed residual (Savitzky–Golay filter; dark gray), plotted for clarity to distinguish Fourier truncation artifacts. From top to bottom, the particle is a simple sphere, a linear chain of pseudo-atoms, a cubic array of pseudo-atoms, and, finally, $[Ru(bpy)_3]^{2+}$. The surface of the van der Waals volume of the corresponding morphology is rendered to the right of each subplot.

same reference trajectory of neat water, we have computed $I_x(Q)$ for $0.5 \leq Q \leq 24$ Å^{-1}, considering excluded solvent volumes defined by these distinct particles, and employing the methodology described above.

In Figure S1, we have transformed these to the "standard" reduced structure functions appropriate for water, which are compared to the (normalized) total scattering reduced structure function of liquid water, computed considering the trajectory as a whole. Judging by these results, the excluded solvent structure function is, to a high degree of precision, identical to the "bulk" structure function for $Q \geq 10$ Å^{-1}. However, as expected, this correspondence begins to deteriorate for $1 < Q < 10$ Å^{-1}, and becomes increasingly poor below $Q \approx 1$ Å^{-1}. Nevertheless, in the HEXS regime ($Q \gtrsim 1$ Å^{-1}), the absolute deviation is relatively small, hence, it may indeed be an adequate approximation to model the excluded solvent by a semi-empirical scaling of the "bulk" structure.

3.3 Evaluating concentration dependence

As mentioned in the main text, we investigated the concentration dependence of our *ansatz* by additionally simulating a 0.5 M aqueous solution of $[Ru(bpy)_3]Cl_2$. This is not a stable solution in reality; however, we found no evidence of crystallization *in silico*, at least over the course of a 20 ns trajectory. For consistency with respect to finite size errors, we simulated this solution by including 32 replicas of the solute, replacing $32 \times 22 = 704$ water molecules from the reference water model.

Figure S2 compares the reduced differential structure functions, and their corresponding Fourier transforms, computed from the 15 mM simulation and the 0.5 M simulation. The residuals do not exhibit any discernible structure, at least within the noise level present in the 15 mM calculation. We conclude that the system behaves as a dilute solution at least up to 0.5 M concentrations, using these force fields.

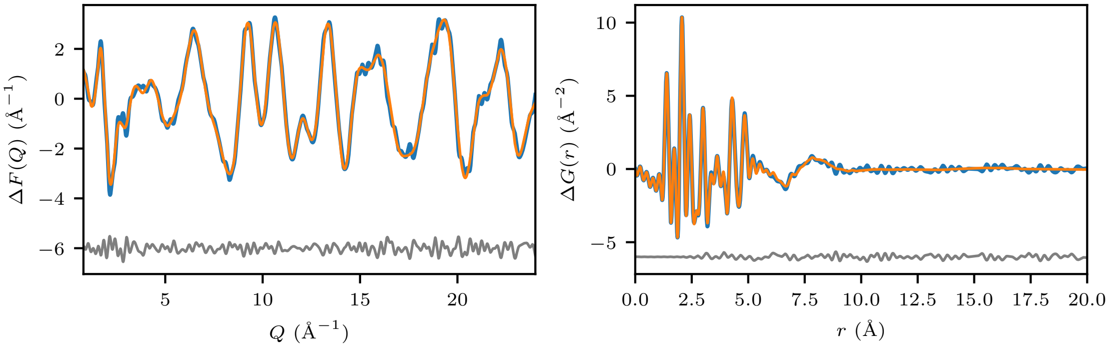

Figure S2 : Comparison of $\Delta F(Q)$ (left) and $\Delta G(r)$ (right; Fourier transformed over $Q \in [0.8, 21.4]$ Å^{-1}), computed from a aqueous simulation of [Ru(bpy)Cl at 15 mM (thick blue lines) and at 0.5 M (thin orange lines). The differences are plotted below in grey.

4 Experimental data reduction

Following correction for dark counts and the detector flat field, each experimental image was calibrated, masked to exclude the beamstop, corrected for the polarization of the X-rays, and integrated azimuthally to yield 1-dimensional scattering curves, $I_A^{(m)}(Q)/I_B^{(m)}(Q)$ over the range $Q_{\min} = 0.6$ and $Q_{\max} = 24$ Å^{-1}; these steps were performed using GSAS-II [22]. Given the experimental configuration of APS beamline 11-ID-B, we do not have access to flux-normalized detector counts, so we do not, in general, obtain absolute scattering intensities, but rather [23],

$$I_x^{(m)}(Q) = \Phi_x(Q)\left[A_x(Q)I_x(Q) + b_x(Q)\right] \Leftrightarrow I_x(Q) = \frac{I_x^{(m)}(Q)}{\Phi_x(Q)A_x(Q)} - \frac{b_x(Q)}{A_x(Q)} \tag{S4.11}$$

where $x = A, B$. $\Phi_x(Q)$ is a normalization term accounting for detector-specific effects (*e.g.*, due to the polarization of the X-rays, detector efficiency, etc.), $A_x(Q)$ is the self-attenuation of the sample, and $b_x(Q)$ is an additive contribution due to other scattering processes (by the experimental apparatus, the container, and any multiple scattering). Having corrected for detector and geometry-specific effects in the azimuthal-integration, $\Phi_x(Q)$ is reduced to a constant, ϕ_x, accounting for any differences in incident flux. We have further corrected the data to account for the self-attenuation and multiple-scattering due to the bulk solvent (water), assuming that the dilute solute in sample A does not introduce significant additional attenuation/multiple scattering. These corrections were performed using the program PDFGetX2 [24], using the theoretical mass-attenuation coefficient for water calculated from the sample dimensions and assuming a packing fraction of unity.

Using these corrected data—labeled (m, corr)—we can approximate the scattering differential by,

$$\begin{aligned}\Delta I(Q) = I_A(Q) - I_B(Q) &\approx \phi_A^{-1} I_A^{(m,\text{corr})}(Q) - \phi_B^{-1} I_B^{(m,\text{corr})}(Q) \\ &= w_0 I_A^{(m,\text{corr})}(Q) + w_1 I_B^{(m,\text{corr})}(Q) \\ &= \boldsymbol{I}^{(m)}\boldsymbol{w}\end{aligned} \tag{S4.12}$$

where $\boldsymbol{I}^{(m)} := \left[I_A^{(m,\text{corr})}, I_B^{(m,\text{corr})}\right]$ and $\boldsymbol{w} := [w_0, w_1]^T$. Here, we assume that any remaining, additive, correction terms are identical between the two measurements, and thereby cancel. To take the proper difference, then, we need a method to estimate the unknown weights, $w_0 := \phi_A^{-1}$ and $w_1 := -\phi_B^{-1}$. To do so, we cast the differential calculation as an optimization problem.

Recall that we can partition all of the atoms in the liquid unit cell of sample A ($\mathcal{A}$, of

which there are $N_{\mathcal{A}}$), into the set of solute atoms, $\mathcal{U}$ (of which there are $N_{\mathcal{U}}$), and the rest. Thus, the *total* scattering intensity from measurement A, in electron units, can be written,

$$\begin{aligned} I_A(Q) &= I^A_{\text{coh}}(Q) + I^A_{\text{inc}}(Q) \\ &= \left[N_{\mathcal{A}} \langle f(Q)^2 \rangle_{\mathcal{A}} + I^A_{\text{d}}(Q)\right] + N_{\mathcal{A}} \langle C(Q) \rangle_{\mathcal{A}} \\ &= \left(\sum_{i \in \mathcal{U}}^{N_{\mathcal{U}}} f_i^*(Q) f_i(Q) + \sum_{i \in \mathcal{A} \setminus \mathcal{U}}^{N_{\mathcal{A}} - N_{\mathcal{U}}} f_i^*(Q) f_i(Q) \right) + I^A_{\text{d}}(Q) + \left(\sum_{i \in \mathcal{U}}^{N_{\mathcal{U}}} C_i(Q) + \sum_{i \in \mathcal{A} \setminus \mathcal{U}}^{N_{\mathcal{A}} - N_{\mathcal{U}}} C_i(Q) \right) \\ &= I^A_{\text{d}}(Q) + N_{\mathcal{U}} \left[\langle f(Q)^2 \rangle_{\mathcal{U}} + \langle C(Q) \rangle_{\mathcal{U}}\right] + \left(\sum_{i \in \mathcal{A} \setminus \mathcal{U}}^{N_{\mathcal{A}} - N_{\mathcal{U}}} f_i^*(Q) f_i(Q) + \sum_{i \in \mathcal{A} \setminus \mathcal{U}}^{N_{\mathcal{A}} - N_{\mathcal{U}}} C_i(Q) \right) \end{aligned} \tag{S4.13}$$

and similarly for $I_B(Q)$, replacing the label $\mathcal{U}$ with the label $\mathcal{E}$ (*i.e.*, identifying the excluded solvent droplet in place of the solute). In the limit of an *ideal* solution (*i.e.*, infinitely dilute), we have $N_{\mathcal{B}} \to (N_{\mathcal{A}} - N_{\mathcal{U}}) + N_{\mathcal{E}}$. Hence, upon taking the differential $\Delta I(Q) = I_A(Q) - I_B(Q)$, the terms in the parentheses of Eqn. (S4.13) identically cancel. We are left, after some rearrangement,

$$\Delta I_{\text{d}}(Q) = \Delta I(Q) - \left(N_{\mathcal{U}} \left[\langle f(Q)^2 \rangle_{\mathcal{U}} + \langle C(Q) \rangle_{\mathcal{U}}\right] - N_{\mathcal{E}} \left[\langle f(Q)^2 \rangle_{\mathcal{E}} + \langle C(Q) \rangle_{\mathcal{E}}\right]\right) \tag{S4.14}$$

That is, the differential distinct scattering is obtained after subtracting the "differential self-scattering" ($\Delta I_{\text{self}}(Q) \coloneqq N_{\mathcal{U}} \langle f(Q)^2 \rangle_{\mathcal{U}} - N_{\mathcal{E}} \langle f(Q)^2 \rangle_{\mathcal{E}}$) and the "differential Compton scattering" ($\Delta I_{\text{inc}}(Q) \coloneqq N_{\mathcal{U}} \langle C(Q) \rangle_{\mathcal{U}} - N_{\mathcal{E}} \langle C(Q) \rangle_{\mathcal{E}}$). Note, however, that—given we have a reasonable estimate for $N_{\mathcal{E}}$—both $\Delta I_{\text{self}}(Q)$ and $\Delta I_{\text{inc}}(Q)$ are calculable, depending on, as they do, composition and not structure.

Now, consider the asymptotic properties of the left hand side of Eqn. (S4.14). The differential distinct scattering is a function which exhibits damped oscillations about zero as $Q \to \infty$. It is, therefore, a function that "eventually" becomes small:

$$\forall\, \varepsilon > 0,\ \exists\, Q_\varepsilon \ \text{ s.t. } \left. \|\Delta I_{\text{d}}(Q)\|_{\ell_2} \right|_{Q_\varepsilon}^{\infty} < \varepsilon \tag{S4.15}$$

Hence, combining Eqns. (S4.12) and (S4.14), we arrive at the convex program,

$$\min_{\boldsymbol{w} \in \mathbb{R}^2} \left. \left\| \boldsymbol{I}^{(m)} \boldsymbol{w} - \left[\Delta I_{\text{self}}(Q) + \Delta I_{\text{inc}}(Q)\right] \right\|_{\ell_2} \right|_{Q_\varepsilon}^{Q_{\max}} \tag{S4.16}$$

which, for a suitably large choice of Q_ε, should return a reasonable estimate for the correct coefficients—provided the data are of high-quality, properly corrected, and that the solution

behaves approximately ideally.

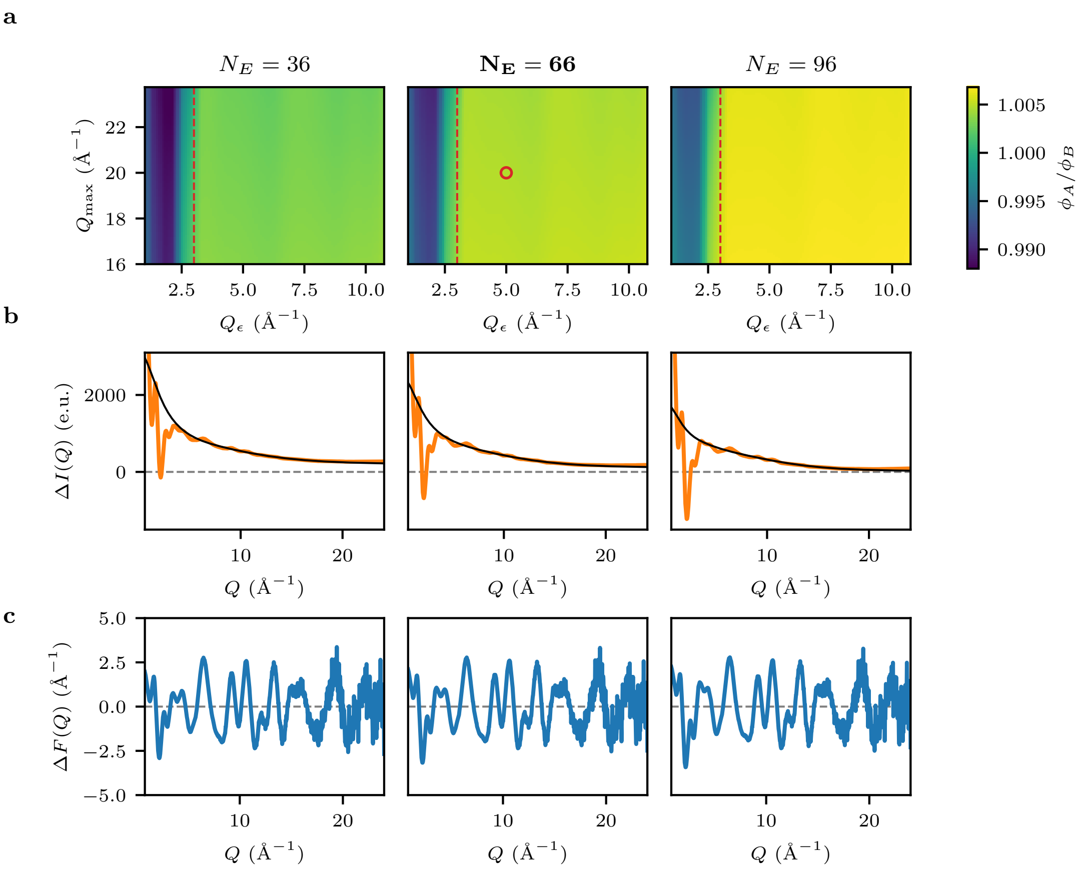

Figure S3 : **a,** Ratio of the optimal scale factors (ϕ_A/ϕ_B) as a function ($Q_\varepsilon, Q_{\max}$), at three fixed values of $N_\mathcal{E}$, as indicated. The circle on the $N_\mathcal{E} = 66$ surface indicates the final values used in further data reduction. **b,** Examples of $\Delta I(Q)$ resulting from program (S4.16) with, from left-to-right, $(N_\mathcal{E}, Q_\varepsilon, Q_{\max}) = (36, 5, 20), (66, 5, 20), (96, 5, 20)$. The solid black lines depict the sum of the calculated $\Delta I_{\text{self}}(Q)$ and $\Delta I_{\text{inc}}(Q)$. **c,** Final reduced data produced from the differentials given above.

We explore the hyperparameter sensitivity of program (S4.16) in Figure S3a. We find that, for a given value of $N_\mathcal{E}$, the ratio of the optimized coefficients (ϕ_A/ϕ_B) is insensitive to the particular choice of $(Q_\varepsilon, Q_{\max})$ above a critical threshold $Q_\varepsilon \gtrsim 3$ Å^{-1} (Fig. 3a, dashed red lines); this roughly coincides with the falling edge of the total scattering arising from neat water (*c.f.* Fig. S4a), the region where the differential scattering should most significantly deviate from zero (in absolute terms). The exact value for this ratio depends on the particular choice of $N_\mathcal{E}$, with typical values 1.003 for $N_\mathcal{E} = 36$ (significantly smaller than our calculated estimate); 1.005 for $N_\mathcal{E} = 66$ (our calculated estimate); and 1.006 for

$N_{\mathcal{E}} = 96$ (significantly larger than our calculated estimate). We expect that too low a value for $N_{\mathcal{E}}$ produces undersubtracted data, while too large a value produces oversubtracted data (Fig. 3b). Nevertheless, as shown in Figure 3c, this sensitivity only manifests in a slight variation in the absolute intensities of features in the low-Q region of the final $\Delta F(Q)$, after further baseline correction (*vide infra*).

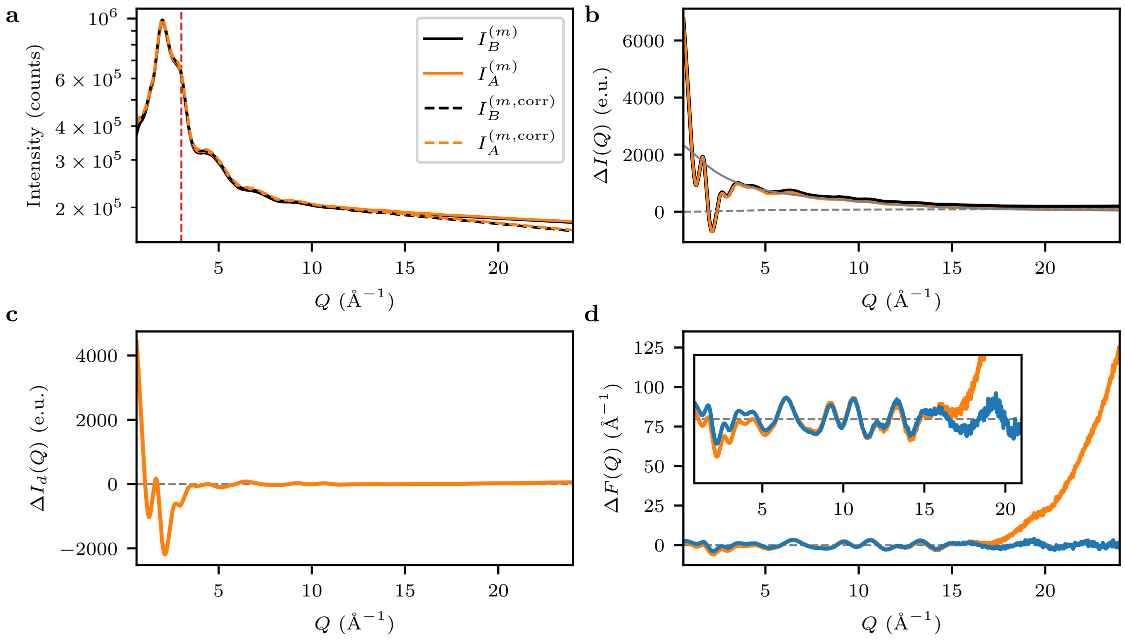

Figure S4 : **a,** Raw, azimuthally-integrated data, $I^{(m)}(Q)$, as well as corrected data using PDFGetX2 [24], $I^{(m,\mathrm{corr})}(Q)$. Data labeled B (black) are from a measurement of neat water, while those labeled A (orange) are from a measurement of 15 mM aqueous $[\mathrm{Ru(bpy)_3}]\mathrm{Cl_2}$. **b,** The differential total scattering following optimization, as described. The grey lines show the differential self-scattering ($\Delta I_{\mathrm{self}}(Q)$, solid line), and the differential Compton scattering ($\Delta I_{\mathrm{inc}}(Q)$, dashed line). **c,** The differential distinct scattering, following subtraction of $\Delta I_{\mathrm{self}}(Q)$ and $\Delta I_{\mathrm{self}}(Q)$. **d,** Transformation of the "raw" $\Delta I_{\mathrm{d}}(Q)$ to the differential total scattering structure function, $\Delta F(Q)$, (orange line), along with the final, baseline-corrected data (blue line).

For the data presented in the main text, we employed $(N_{\mathcal{E}}, Q_{\varepsilon}, Q_{\mathrm{max}}) = (66, 5, 20)$. The complete reduction to the differential reduced structure function is depicted in Figure S4. We note that, after transforming the "raw" $\Delta I_{\mathrm{d}}(Q)$ (Fig. S4c) to $\Delta F(Q)$, the structure function oscillates about zero, as expected, but begins to diverge smoothly for $Q \gtrsim 17$ Å^{-1} (Fig. S4d, orange line). There are a number of potential causes for this errant behavior, including improper data corrections, improper flat-field correction of the detector [25], and, perhaps most expectedly, inaccurate calculation of the differential Compton scattering.

Nevertheless, employing the *ad hoc* baseline correction algorithm published by Juhás, *et al.* [26], we obtain the final $\Delta F(Q)$ shown in Figure S4d (blue line). While this *ad hoc* procedure may itself introduce spurious signals into the data, the final comparison between our experimental results and the *ab initio* simulation validates the data reduction *ansatz* developed here.

Supplementary References

(1) Frisch, M. J.; Trucks, G. W.; Schlegel, H. B.; Scuseria, G. E.; Robb, M. A.; Cheeseman, J. R.; Scalmani, G.; Barone, V.; Petersson, G. A.; Nakatsuji, H.; Li, X.; Caricato, M.; Marenich, A. V.; Bloino, J.; Janesko, B. G.; Gomperts, R.; Mennucci, B.; Hratchian, H. P.; Ortiz, J. V.; Izmaylov, A. F.; Sonnenberg, J. L.; Williams-Young, D.; Ding, F.; Lipparini, F.; Egidi, F.; Goings, J.; Peng, B.; Petrone, A.; Henderson, T.; Ranasinghe, D.; Zakrzewski, V. G.; Gao, J.; Rega, N.; Zheng, G.; Liang, W.; Hada, M.; Ehara, M.; Toyota, K.; Fukuda, R.; Hasegawa, J.; Ishida, M.; Nakajima, T.; Honda, Y.; Kitao, O.; Nakai, H.; Vreven, T.; Throssell, K.; Montgomery Jr., J. A.; Peralta, J. E.; Ogliaro, F.; Bearpark, M. J.; Heyd, J. J.; Brothers, E. N.; Kudin, K. N.; Staroverov, V. N.; Keith, T. A.; Kobayashi, R.; Normand, J.; Raghavachari, K.; Rendell, A. P.; Burant, J. C.; Iyengar, S. S.; Tomasi, J.; Cossi, M.; Millam, J. M.; Klene, M.; Adamo, C.; Cammi, R.; Ochterski, J. W.; Martin, R. L.; Morokuma, K.; Farkas, O.; Foresman, J. B.; Fox, D. J. Gaussian 16 Revision C.01, Gaussian Inc. Wallingford CT, 2016.

(2) Staroverov, V. N.; Scuseria, G. E.; Tao, J.; Perdew, J. P. Comparative assessment of a new nonempirical density functional: Molecules and hydrogen-bonded complexes. *The Journal of Chemical Physics* **2003**, *119*, 12129–12137, DOI: 10.1063/1.1626543.

(3) Weigend, F.; Ahlrichs, R. Balanced basis sets of split valence, triple zeta valence and quadruple zeta valence quality for H to Rn: Design and assessment of accuracy. *Phys. Chem. Chem. Phys.* **2005**, *7*, 3297–3305, DOI: 10.1039/B508541A.

(4) Singh, U. C.; Kollman, P. A. An approach to computing electrostatic charges for molecules. *Journal of Computational Chemistry* **1984**, *5*, 129–145, DOI: https://doi.org/10.1002/jcc.540050204.

(5) Besler, B. H.; Merz Jr., K. M.; Kollman, P. A. Atomic charges derived from semiempirical methods. *Journal of Computational Chemistry* **1990**, *11*, 431–439, DOI: https://doi.org/10.1002/jcc.540110404.

(6) Rappe, A. K.; Casewit, C. J.; Colwell, K. S.; Goddard, W. A. I.; Skiff, W. M. UFF, a full periodic table force field for molecular mechanics and molecular dynamics simulations. *Journal of the American Chemical Society* **1992**, *114*, 10024–10035, DOI: 10.1021/ja00051a040.

(7) Li, P.; Merz, K. M. J. MCPB.py: A Python Based Metal Center Parameter Builder. *Journal of Chemical Information and Modeling* **2016**, *56*, 599–604, DOI: 10.1021/acs.jcim.5b00674.

(8) Case, D. A.; Aktulga, H. M.; Belfon, K.; Ben-Shalom, I. Y.; Brozell, S. R.; Cerutti, D. S.; Cheatham III, T. E.; Cisneros, G. A.; Cruzeiro, V. W. D.; Dardenk, T. A.; Duke, R. E.; Giambasu, G.; Gilson, M. K.; Gohlke, H.; Goetz, A. W.; Harris, R.; Izadi, S.; Izmailov, S. A.; Jin, C.; Kasavajhala, K.; Kaymak, M. C.; King, E.; Kovalenko, A.; Kurtzman, T.; Lee, T. S.; LeGrand, S.; Li, P.; Lin, C.; Liu, J.; Luchko, T.; Luo, R.; Machado, M.; Man, V.; Manathunga, M.; Merz, K. M.; Miao, Y.; Mikhailovskii, O.; Monard, G.; Nguyen, H.; O'Hearn, K. A.; Onufriev, A.; Pan, F.; Pantano, S.; Qi, R.; Rahnamoun, A.; Roe, D. R.; Roitberg, A.; Sagui, C.; Schott-Verdugo, S.; Shen, J.; Simmerling, C. L.; Skrynnikov, N. R.; Smith, J.; Swails, J.; Walker, R. C.; Wang, J.; Wei, H.; Wolf, R. M.; Wu, X.; Xue, Y.; York, D. M.; Zhao, S.; Kollman, P. A. Amber2021, University of California, San Francisco, 2021.

(9) Izadi, S.; Anandakrishnan, R.; Onufriev, A. V. Building Water Models: A Different Approach. *The Journal of Physical Chemistry Letters* **2014**, *5*, 3863–3871, DOI: 10.1021/jz501780a.

(10) Sengupta, A.; Li, Z.; Song, L. F.; Li, P.; Merz, K. M. J. Parameterization of Monovalent Ions for the OPC3, OPC, TIP3P-FB, and TIP4P-FB Water Models. *Journal of Chemical Information and Modeling* **2021**, *61*, 869–880, DOI: 10.1021/acs.jcim.0c01390.

(11) Eastman, P.; Swails, J.; Chodera, J. D.; McGibbon, R. T.; Zhao, Y.; Beauchamp, K. A.; Wang, L.-P.; Simmonett, A. C.; Harrigan, M. P.; Stern, C. D.; Wiewiora, R. P.; Brooks, B. R.; Pande, V. S. OpenMM 7: Rapid development of high performance algorithms for molecular dynamics. *PLOS Computational Biology* **2017**, *13*, 1–17, DOI: 10.1371/journal.pcbi.1005659.

(12) Dohn, A. O.; Biasin, E.; Haldrup, K.; Nielsen, M. M.; Henriksen, N. E.; Møller, K. B. On the calculation of x-ray scattering signals from pairwise radial distribution functions. *Journal of Physics B: Atomic, Molecular and Optical Physics* **2015**, *48*, 244010, DOI: 10.1088/0953-4075/48/24/244010.

(13) Warren, B. E., *X-ray Diffraction*; Dover Publications: New York, 1990.

(14) Neder, R. B.; Proffen, T. Exact and fast calculation of the X-ray pair distribution function. *Journal of Applied Crystallography* **2020**, *53*, 710–721, DOI: 10.1107/S1600576720004616.

(15) Dohn, A. O.; Markmann, V.; Nimmrich, A.; Haldrup, K.; Møller, K. B.; Nielsen, M. M. Eliminating finite-size effects on the calculation of x-ray scattering from molecular dynamics simulations. *The Journal of Chemical Physics* **2023**, *159*, 124115, DOI: 10.1063/5.0164365.

(16) Juhás, P.; Farrow, C. L.; Yang, X.; Knox, K. R.; Billinge, S. J. L. Complex modeling: a strategy and software program for combining multiple information sources to solve ill posed structure and nanostructure inverse problems. *Acta Crystallographica Section A* **2015**, *71*, 562–568, DOI: 10.1107/S2053273315014473.

(17) Waasmaier, D.; Kirfel, A. New analytical scattering-factor functions for free atoms and ions. *Acta Crystallographica Section A* **1995**, *51*, 416–431, DOI: 10.1107/S0108767394013292.

(18) McGibbon, R. T.; Beauchamp, K. A.; Harrigan, M. P.; Klein, C.; Swails, J. M.; Hernández, C. X.; Schwantes, C. R.; Wang, L.-P.; Lane, T. J.; Pande, V. S. MDTraj: A Modern Open Library for the Analysis of Molecular Dynamics Trajectories. *Biophysical Journal* **2015**, *109*, 1528 –1532, DOI: 10.1016/j.bpj.2015.08.015.

(19) Egami, T.; Billinge, S. J., *Underneath the Bragg Peaks: Structural Analysis of Complex Materials*, 2nd ed.; Pergamon: New York, 2012.

(20) Thijsse, B. J. The accuracy of experimental radial distribution functions for metallic glasses. *Journal of Applied Crystallography* **1984**, *17*, 61–76, DOI: 10.1107/S002188988401102X.

(21) Cromer, D. T.; Mann, J. B. Compton Scattering Factors for Spherically Symmetric Free Atoms. *The Journal of Chemical Physics* **1967**, *47*, 1892–1893, DOI: 10.1063/1.1712213.

(22) Toby, B. H.; Von Dreele, R. B. *GSAS-II*: the genesis of a modern open-source all purpose crystallography software package. *Journal of Applied Crystallography* **2013**, *46*, 544–549, DOI: 10.1107/S0021889813003531.

(23) Peterson, P. F.; Olds, D.; McDonnell, M. T.; Page, K. Illustrated formalisms for total scattering data: a guide for new practitioners. *Journal of Applied Crystallography* **2021**, *54*, 317–332, DOI: 10.1107/S1600576720015630.

(24) Qiu, X.; Thompson, J. W.; Billinge, S. J. L. *PDFgetX2*: a GUI-driven program to obtain the pair distribution function from X-ray powder diffraction data. *Journal of Applied Crystallography* **2004**, *37*, 678, DOI: 10.1107/S0021889804011744.

(25) Weng, J.; Xu, W.; Wiaderek, K. M.; Borkiewicz, O. J.; Chen, J.; Von Dreele, R. B.; Gallington, L. C.; Ruett, U. *In situ* X-ray area detector flat-field correction at an operating photon energy without flat illumination. *Journal of Synchrotron Radiation* **2023**, *30*, 546–554, DOI: 10.1107/S1600577523001157.

(26) Juhás, P.; Davis, T.; Farrow, C. L.; Billinge, S. J. L. *PDFgetX3*: a rapid and highly automatable program for processing powder diffraction data into total scattering pair distribution functions. *Journal of Applied Crystallography* **2013**, *46*, 560–566, DOI: 10.1107/S0021889813005190.
